# Targeted metabolomics identifies high performing diagnostic and prognostic biomarkers for COVID-19

**DOI:** 10.1038/s41598-021-94171-y

**Published:** 2021-07-19

**Authors:** Yamilé López-Hernández, Joel Monárrez-Espino, Ana-Sofía Herrera-van Oostdam, Julio Enrique Castañeda Delgado, Lun Zhang, Jiamin Zheng, Juan José Oropeza Valdez, Rupasri Mandal, Fátima de Lourdes Ochoa González, Juan Carlos Borrego Moreno, Flor M. Trejo-Medinilla, Jesús Adrián López, José Antonio Enciso Moreno, David S. Wishart

**Affiliations:** 1 Cátedras-CONACyT, Consejo Nacional de Ciencia y Tecnologia, 03940 México, México; 2 Autonomous University of Zacatecas, 98000 Zacatecas, Mexico; 3 Christus Muguerza Hospital Chihuahua-University of Monterrey, 31000 Chihuahua, Mexico; 4 Faculty of Medicine, Autonomous University of San Luis Potosí, 78210 San Luis Potosi, Mexico; 5 Unidad de Investigación Biomédica de Zacatecas, Instituto Mexicano del Seguro Social, 98000 Zacatecas, México; 6 The Metabolomics Innovation Center, University of Alberta, Edmonton, AB T6G1C9 Canada; 7 Doctorado en Ciencias Básicas, Universidad Autónoma de Zacatecas, Zacatecas, México; 8 Departmento de Epidemiología, Hospital General de Zona #1 “Emilio Varela Luján”, Instituto Mexicano del Seguro Social, 98000 Zacatecas, México; 9 MicroRNAs Laboratory, Academic Unit for Biological Sciences, Autonomous University of Zacatecas, 98000 Zacatecas, Mexico

**Keywords:** Biochemistry, Chemical biology, Computational biology and bioinformatics, Microbiology, Biomarkers

## Abstract

Research exploring the development and outcome of COVID-19 infections has led to the need to find better diagnostic and prognostic biomarkers. This cross-sectional study used targeted metabolomics to identify potential COVID-19 biomarkers that predicted the course of the illness by assessing 110 endogenous plasma metabolites from individuals admitted to a local hospital for diagnosis/treatment. Patients were classified into four groups (≈ 40 each) according to standard polymerase chain reaction (PCR) COVID-19 testing and disease course: PCR−/controls (i.e., non-COVID controls), PCR+/not-hospitalized, PCR+/hospitalized, and PCR+/intubated. Blood samples were collected within 2 days of admission/PCR testing. Metabolite concentration data, demographic data and clinical data were used to propose biomarkers and develop optimal regression models for the diagnosis and prognosis of COVID-19. The area under the receiver operating characteristic curve (AUC; 95% CI) was used to assess each models’ predictive value. A panel that included the kynurenine: tryptophan ratio, lysoPC a C26:0, and pyruvic acid discriminated non-COVID controls from PCR+/not-hospitalized (AUC = 0.947; 95% CI 0.931–0.962). A second panel consisting of C10:2, butyric acid, and pyruvic acid distinguished PCR+/not-hospitalized from PCR+/hospitalized and PCR+/intubated (AUC = 0.975; 95% CI 0.968–0.983). Only lysoPC a C28:0 differentiated PCR+/hospitalized from PCR+/intubated patients (AUC = 0.770; 95% CI 0.736–0.803). If additional studies with targeted metabolomics confirm the diagnostic value of these plasma biomarkers, such panels could eventually be of clinical use in medical practice.

## Introduction

The severe acute respiratory syndrome coronavirus 2 (SARS-CoV-2), first identified in China in December 2019, is responsible for the coronavirus disease outbreak (COVID-19)^1^. One year after the pandemic was declared, the infection has caused nearly 2.5 million deaths worldwide^2^. In spite of significant efforts undertaken by government and health authorities to contain the spread, the virus continues to wreak havoc around the world.

Since COVID-19 can lead to multi-organ dysfunction, disease severity is not only the result of pathogen burden^3^, but also the consequence of the host’s immune response to the infection. It is well known that viruses hijack the host cell machinery for self-replication, as they compete for nutrients and other metabolites to satisfy their bioenergetic and biosynthetic requirements. This metabolic hijacking can lead to an alteration of the host’s metabolome^4^. In fact, a number of metabolic pathways have already been found to be consistently altered (glycolysis, fatty acid synthesis, glutaminolysis, pyrimidine metabolism, and tryptophan/kynurenine metabolism) in many viral infections (cytomegalovirus, hepatitis C, herpes simplex, chikungunya, and dengue). These alterations in metabolic pathways leading to subsequent changes in metabolite concentrations^5^.

In the early phase of the pandemic, COVID-19 diagnosis faced many technical and logistic difficulties due to the poor sensitivity and specificity of some of the diagnostic techniques used^6^. However, since then more reliable point-of-care diagnostic, therapeutic, and prognostic tools have been developed and implemented. Unfortunately, there are essentially no tests that can be reliably stage or predict/prognose the outcome or the severity of the disease. Given the wide range of outcomes (asymptomic disease to death, rapid recovery to long-haul morbidities) it is important that better algorithms for disease staging, and better prognostic tests be developed. Ideally, what is needed is a test that can rapidly and accurately: (a) distinguish COVID-19 positive from COVID-19 negative patients (despite similar symptoms); (b) prognose or predict those who will develop mild COVID-19 symptoms vs. those who will develop severe COVID-19 symptoms; and (c) distinguish those with severe-COVID who will recover and those who will die. It is in this context that we believe metabolomics could play a valuable role in identifying potential disease staging or prognostic biomarkers that could be applied in clinical practice to assist medical decisions throughout the patients’ care journey.

Studies with COVID-19 patients using untargeted or targeted metabolomics have consistently shown increased levels of glucose, free fatty acids, sphingomyelins and triglycerides, as well as changes in serum tryptophan metabolism^7–12^. As a result, various potential diagnostic and prognostic biomarkers have been proposed. For instance, Barberis et al.^12^ proposed the use of seven lipids and eight non-lipid metabolites in plasma to discriminate COVID-19 patients from healthy individuals, with a receiver operating characteristic (ROC) area under the curve (AUC) ˃ 0.90 for such model. Similarly, Song et al.^13^ also proposed a panel of ten plasma metabolites to distinguish COVID-19 patients from healthy controls with an AUC ˃ 0.90. On the other hand, Fraser et al.^14^ reported that plasma kynurenine and the arginine: kynurenine ratio gave a 98% classification accuracy (p = 0.005) to distinguish COVID-19 positive from COVID-19 negative patients, while creatinine and the creatinine: arginine ratio gave a 100% accuracy when predicting COVID-19 mortality (p = 0.01). More recently, Danlos et al.^15^ found at least 77 metabolites including amino acids, lipids, polyamines and sugars, as well as their derivatives, that were altered in critical COVID-19 patient’s plasma as compared to mild COVID-19 patients^15^. As general findings among the published works up to now conducted in different populations, it seems that the SARS-CoV-2 virus led to stimulated systemic inflammatory and reduced liver synthesis capacity in COVID-19 subjects^16^. Plasma metabolomics reflect the disturbed amino acid metabolism and increased levels of acylcarnitine related metabolites, as consequence of the impaired fatty acid oxidation, also demonstrated by Wu et al.^17^ and Li et al.^18^. However, given the small size of these studies and the lack of follow-up validation studies, much more still needs to be done to produce useful and validated biomarkers that could adequately serve the medical community.

The study described here used targeted metabolomics to identify potential biomarkers to: (a) distinguish COVID-19 negative from COVID-19 positive patients with mild disease; (b) prognose or predict those who will develop mild COVID-19 symptoms vs. those who will develop severe COVID-19 symptoms; (c) prognose those severe COVID-19 patients who will be intubated; and (d) find a metabolite panel can distinguish those with severe COVID-19 who will recover and those who will die. We proposed different biomarker panels based on metabolites-only and metabolites plus demographic/clinical data with high predictive values. This was done by assessing 110 endogenous metabolites in plasma collected from individuals admitted to a local hospital for initial diagnosis/treatment of a presumptive COVID-19 infection. Patients were classified into four groups (≈ 40 each) according to standard polymerase chain reaction (PCR) COVID-19 testing and disease course/outcomes: G1 (PCR−/controls), G2 (PCR+/not hospitalized), G3 (PCR+/ hospitalized), and G4 (PCR+/intubated). This classification was used as a surrogate of disease severity. Blood samples were collected simultaneously with the nasopharyngeal sampling, within 2 days of time of admission and prior to any knowledge of the COVID-19 diagnosis or disease outcome. Metabolites were measured using a locally developed LC–MS/MS metabolomics assay called The Metabolomics Innovation Centre (TMIC) Prime (TMIC PRIME®) Assay. This assay was adapted to work with plasma using a similar quantitative assay developed for urine^19^. TMIC PRIME® provides quantitative results for up to 143 endogenous metabolites, including biogenic amines, amino acids, organic acids and lipid-like compounds.

## Results

### Patients

Table 1 describes the study population stratified according to our COVID-19 severity classification. There was a higher proportion of males in groups 2 and 3 (57%), but especially in group 4 (62%), compared with group 1 (45%). The median age was also notably higher in groups 2–4 (53–58 years) compared with that of group 1 (41 years). General and respiratory symptoms of COVID-19 infection tended to be more prevalent as the group number increased. Diabetes, obesity and hypertension were also more prevalent among patients from groups 3 and 4, with group 4 patients having an incidence of these three conditions of 27.5%, 32.5% and 50%, respectively. Laboratory data also tended to reflect the severity of the disease according to the group categorization, with higher levels of neutrophilia, lymphopenia, and hyperglycemia among patients in the higher group numbers. The median time from symptoms onset to sampling was higher in group 4 (5 days) than in group 3 (3 days). Also, the median time to discharge was higher in group 4 (14 days). From the total number of COVID-19 patients (122 patients), 32.8 % required mechanical ventilation. From this group, 63.8 % of patients died. Based on the confirmed data available in medical files at the moment of the present manuscript, 46% of the hospitalized patients did not survive. Supplementary Table 1 describes some relevant clinical data from survivors and non-survivors from the groups 3 and 4.

**Table 1:** Baseline characteristics of non-COVID-19 and COVID-19 patients at admission.

Variables	G1 (N = 39)	G2 (N = 40)	G3 (N = 42)	G4 (N = 40)	p value
Male sex, n (%)	18 (45.0)	23 (57.5)	24 (57.1)	25 (62.5)	0.4
Age, median years (Q1–Q3)	41 (37–53)	58 (51–63)	53 (47–61)	58 (49–62)	< 0.0001^a^
Smoking, n (%)	4 (10.0)	3 (7.5)	6 (14.3)	NA	0.1
Symptoms to sampling, median days (Q1–Q3)	2 (1–5)	3 (1–3)	3 (1–5)	5 (2–7)	0.01^b^
Sampling to discharge, median days (Q1–Q3)	NA	NA	4 (1–10)	14 (7–21)	0.002
Sampling to death, median days (Q1–Q3)	NA	NA	17 (14–25)	10 (6–18)	0.1
Death^2^, n (%)	NA	NA	11/36 (30.5)	21/33 (63.6)	< 0.0001
**Symptomatology, n (%)**					
Fever	0 (0)	22 (55.0)	25 (59.5)	25 (62.5)	< 0.0001
Cough	0 (0)	28 (70.0)	34 (80.9)	36 (90.0)	< 0.0001
Headache	29 (72.5)	30 (75.0)	24 (57.1)	25 (62.5)	0.2
Dyspnoea	5 (12.5)	12 (30.0)	38 (90.4)	31 (77.5)	< 0.0001
Irritability	3 (7.5)	2 (5.0)	3 (7.1)	2 (5.0)	0.9
Diarrhea	2 (5.0)	4 (10.0)	7 (16.6)	7 (17.5)	0.2
Chest tightness	2 (5.0)	6 (15.0)	16 (38.0)	11 (27.5)	0.001
Chills	5 (12.5)	14 (35.0)	17 (40.4)	16 (40.0)	0.02
Pharyngalgia	17 (42.5)	14 (35.0)	14 (33.3)	18 (45.0)	0.6
Myalgia	15 (37.5)	21 (52.5)	23 (54.7)	23 (57.5)	0.2
Arthralgias	11 (27.5)	22 (55.0)	22 (52.3)	21 (52.5)	0.04
Rhinorrhea	6 (15)	8 (20.0)	7 (16.6)	6 (15.0)	0.9
Polypnea	1 (2.5)	0 (0)	7 (16.6)	7 (17.5)	0.006
Abdominal pain	4 (10)	3 (7.5)	2 (4.7)	8 (20.0)	0.1
Anosmya	0 (0)	10 (25.0)	10 (23.8)	3 (7.5)	0.001
Dysgeusia	0 (0)	10 (25.0)	9 (21.4)	5 (12.5)	0.007
**Treatment (self-reported)**					
Antipyretics, n (%)	4 (10.2)	12 (30)	11 (26.2)	13 (32.5)	0.04
Others, n (%)	0 (0)	0 (0)	0 (0)	0 (0)	–
**Comorbidities (self-reported), n (%)**					
Diabetes	3 (7.5)	4 (10.0)	18 (42.8)	11 (27.5)	0.0002
Obesity (> 30 kg/m^2^)	3 (7.5)	8 (20.0)	10 (23.8)	13 (32.5)	0.05
Hypertension	9 (22.5)	10 (25.0)	15 (35.7)	20 (50.0)	0.03
**Lab data, median (Q1–Q3)**					
Erythrocytes (million/mL)	5.1 (4.8–5.5)	5.3 (4.9–5.6)	5.1 (4.7–5.5)	5.3 (4.6–5.6)	0.81
Hemoglobin (g/dL)	15.4 (14.4–16.3)	15.3 (14.0–16.1)	15.0 (12.9–16.6)	15.6 (13.4–16.4)	0.81
Platelets (thousands/mL)	277 (238–330)	237 (191–317)	237 (187–283)	252 (187–299)	0.7
Leukocytes (× 10^3^)	7.1 (6.0–8.2)	6.5 (5.0–8.4)	9.1 (6.5–10.8)	9.3 (6.3–12.2)	0.0005^c^
Neutrophils (%)	60 (54–65)	67 (55–79)	82 (74–88)	85 (77–91)	< 0.0001^d^
Lymphocytes (%)	30 (25–36)	23 (13–34)	12 (7–18)	9 (5–12)	< 0.0001^d^
Neutrophil–lymphocyte ratio (NLR)	2.0 (1.5–2.5)	2.5 (1.5–4.2)	6.6 (3.9–10.7)	7.8 (5.0–14.6)	< 0.0001^d^
Monocytes (%)	6 (5–8)	6 (4–9)	3 (2–6)	3 (2–5)	< 0.0001^e^

Continuous variables were compared using Mann–Whitney U tests or Kruskal–Wallis tests and categorical variables (sex, smoking, death, symptoms, and comorbidities) were compared using the chi-square test for trend, with p values of less than 0.05 considered statistically significant. The analyses were conducted using GraphPad Prism version 8.0.1 for Windows (GraphPad Software, La Jolla California USA).

^a^G1 vs. G2; G1 vs. G3; G1 vs. G4.

^b^G1 vs. G4.

^c^G1 vs. G4; G2 vs. G3; G2 vs. G4.

^d^G1 vs. G3; G1 vs. G4; G2 vs. G3; G2 vs. G4.

^e^G1 vs. G3; G1 vs. G4; G2 vs. G4.

^1^G1: PCR−/controls, G2: PCR+/not hospitalized, G3: PCR+/hospitalized, and G4: PCR+/intubated.

^2^The number (n) is referred to the confirmed data based on clinical and epidemiological records and supported by death certificate.

### Models to distinguish COVID-19 negative from COVID-19 positive patients with mild disease

**Figure 1:**
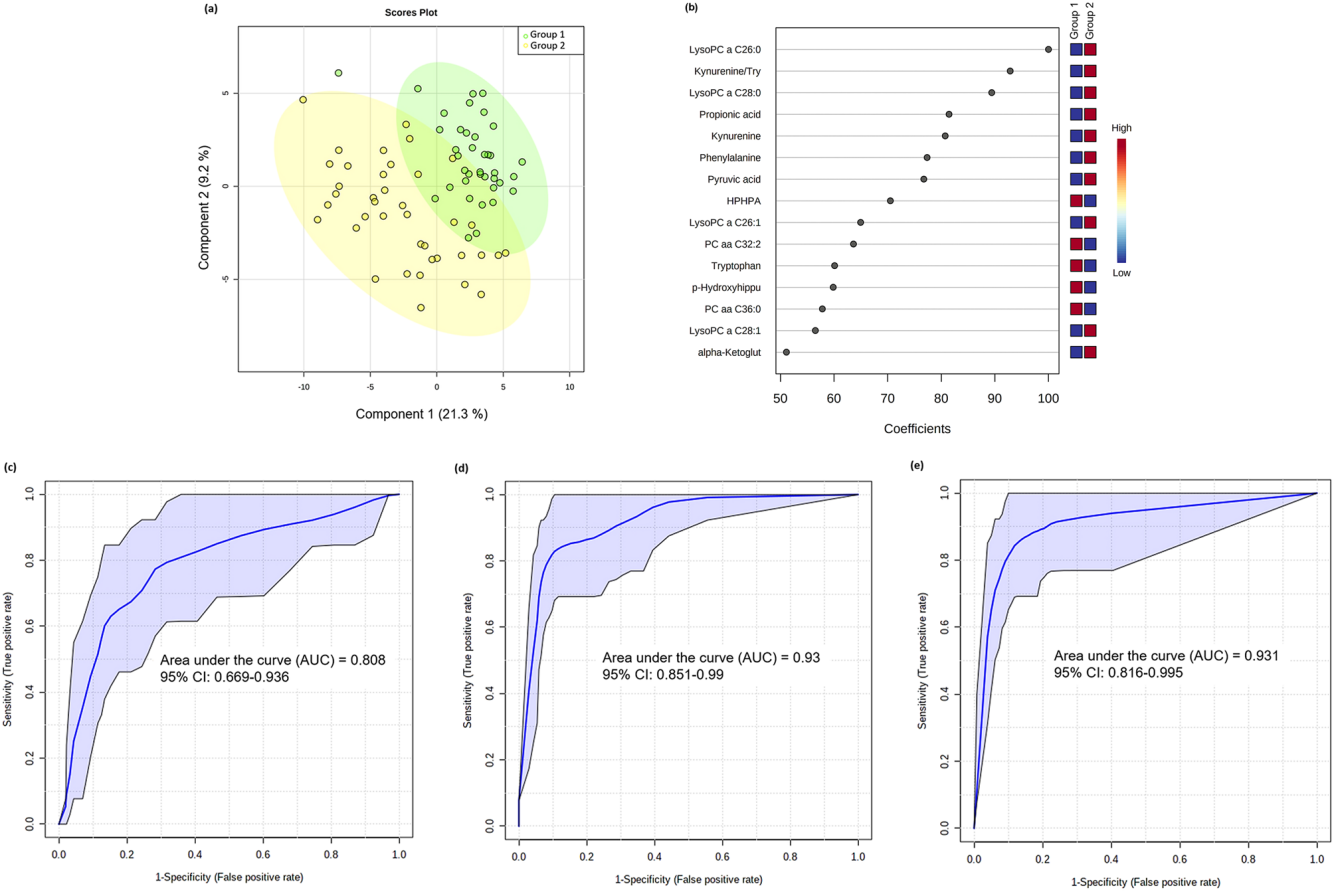
Multivariate analysis from plasma metabolome profile of G1 versus G2 patients. (**a**) Score scatter plot based on the PLS-DA models to explain the diagnosis (green for G1 and yellow for G2; (**b**) rank of the different metabolites (the top 15) identified by the PLS-DA according to the VIP coefficient on the x-axis. The most discriminating metabolites are shown in descending order of their coefficient scores. The color boxes indicate whether metabolite concentration is increased (red) or decreased (blue) in G1 vs G2; (**c**) ROC curve of the demographic/clinical data model; (**d**) ROC curve of the metabolite-only model; (**e**) ROC curve of the metabolite + demographic/clinical data model. The figures were drawn via MetaboAnalyst software v 4.0 (https://www.metaboanalyst.ca/).

The metabolomics comparison between G1 and G2 is shown in Fig 1. Multivariate PLS-DA showed two defined clusters for groups 1 and 2 (accuracy = 0.88, R^2^ = 0.86, Q^2^ = 0.5, permutation test: p < 0.0001) (Fig. 1a); lysoPC a C26:0, kynurenine: tryptophan ratio, lysoPC a C28:0, and propionic acid had the highest scores driving the cluster separation (Fig. 1b). The ROC curve for demographic/clinical data model (discovery set) with 95% confidence interval (CI) is shown in Fig. 1c. The AUC, sensitivity and specificity values with 95% CI are shown in Table 2. A logistic regression model was then built with the following equation: log(P/(1 − P)) = − 0.098 − 1.22 age + 0.722 lymphocytes (%), where the numeric value of each named metabolite in the equation is the concentration after log transformation and auto-scaling.

**Table 2:** The AUC, Sensitivity and Specificity with 95% confidence intervals (CI) of each predictive panel of plasma metabolites (metabolites-only and metabolites plus demographic/clinical data models) for COVID-19.

Predictive models	AUC, 95% CI	Sensitivity, 95% CI	Specificity, 95% CI
**G1 vs G2**			
Demographic/clinical data			
Age + lymphocyte (%)	0.825 (0.794–0.856)^a^	0.830 (0.791–0.870)^a^	0.736 (0.691–0.782)^a^
	0.797 (0.694–0.900)^b^	0.816 (0.816–0.939)^b^	0.725 (0.587–0.863)^b^
Metabolites-only			
Kynurenine/tryptophan + lysoPC a C26:0 + pyruvic acid	0.947 (0.931–0.962)^a^	0.865 (0.829–0.902)^a^	0.897 (0.866–0.929)^a^
	0.922 (0.863–0.981)^b^	0.868 (0.868–0.976)^b^	0.900 (0.807–0.993)^b^
Metabolites + demographic/clinical data			
Kynurenine/tryptophan + lysoPC a C26:0 + pyruvic acid + sex + neutrophil (%)	0.971 (0.960–0.981)^a^	0.930 (0.903–0.957)^a^	0.894 (0.863–0.926)^a^
	0.924 (0.861–0.986)^b^	0.895 (0.895–0.992)^b^	0.875 (0.773–0.977)^b^
**G2 vs G3 + G4**			
Demographic/clinical data			
Lymphocytes (%) + neutrophils (%) + diabetes	0.855 (0.833–0.877)^a^	0.748 (0.717–0.779)^a^	0.808 (0.768–0.849)^a^
	0.826 (0.749–0.904)^b^	0.756 (0.756–0.849)^b^	0.800 (0.676–0.924)^b^
Metabolites only			
C10:2 + butyric acid + pyruvic acid	0.975 (0.968–0.983)^a^	0.962 (0.948–0.976)^a^	0.872 (0.838–0.907)^a^
	0.967 (0.938–0.996)^b^	0.951 (0.951–0.998)^b^	0.875 (0.773–0.977)^b^
Metabolites + demographic/clinical data			
C10:2 + butyric acid + pyruvic acid + lymphocytes (%) + neutrophils (%)	0.989 (0.985–0.993)^a^	0.881 (0.857–0.904)^a^	0.967 (0.948–0.985)^a^
	0.975 (0.953–0.997)^b^	0.878 (0.878–0.949)^b^	0.925 (0.843–1.000)^b^
**G3 vs G4**			
Demographic/clinical data			
NLR	0.653 (0.614–0.692)^a^	0.644 (0.595–0.694)^a^	0.598 (0.548–0.647)^a^
	0.624 (0.501–0.747)^b^	0.650 (0.650–0.798)^b^	0.619 (0.472–0.766)^b^
Metabolites only			
LysoPC a 28:0	0.770 (0.736–0.803)^a^	0.800 (0.759–0.841)^a^	0.638 (0.589–0.686)^a^
	0.764 (0.660–0.868)^b^	0.825 (0.825–0.943)^b^	0.643 (0.498–0.788)^b^
Metabolites + demographic/clinical data			
LysoPC a 28:0 + hypertension + NLR	0.829 (0.800–0.858)^a^	0.775 (0.732–0.818)^a^	0.722 (0.677–0.767)^a^
	0.801 (0.704–0.897)^b^	0.775 (0.775–0.904)^b^	0.762 (0.633–0.891)^b^
**Severe (G3 + G4) vs risk of death (G3 + G4)**			
Demographic/clinical data*			
–	–	–	–
Metabolites only			
LysoPC a 16:0	0.689 (0.649–0.728)^a^	0.656 (0.612–0.699)^a^	0.635 (0.580–0.691)^a^
	0.667 (0.544–0.791)^b^	0.660 (0.660–0.791)^b^	0.656 (0.492–0.821)^b^
Metabolites + demographic/clinical data			
LysoPC a 16:0 + age	0.716 (0.676–0.756)^a^	0.740 (0.699–0.781)^a^	0.656 (0.601–0.711)^a^
	0.691 (0.564–0.818)^b^	0.740 (0.740–0.862)^b^	0.656 (0.492–0.821)^b^

G1: PCR−, controls, G2: PCR+, not hospitalized; G3: PCR+, hospitalized, G4: PCR+, intubated, G3 + G4: severe patients.

*Variables were not significant to be included in the model.

The ROC curve for metabolite-only model (discovery set) with 95% confidence interval (CI) is shown in Fig. 1d. The AUC, sensitivity and specificity values with 95% CI are shown in Table 2. A logistic regression model was then built with the following equation: logit(P) = log(P/(1 − P)) = − 0.444 − 2.599 kynurenine/tryptophan − 1.483 lysoPC a C26:0 − 1.142 pyruvic acid, where the numeric value of each named metabolite in the equation is the concentration after log transformation and auto-scaling.

When sex and neutrophil count (%) were added to the model (Fig. 1e), the AUC was slightly superior to the metabolite-only model (Table 2). The equation for the logistic regression model is: logit(P) = log(P/(1 − P)) = − 0.874 − 3.659 kynurenine/tryptophan − 2.168 lysoPC a C26:0 − 1.703 pyruvic acid + 1.05 sex + 1.179 neutrophils (%). Age and lymphocyte counts did not significantly contribute when combined to metabolite measurements in the logistic regression model.

Additional details for demographic/clinical data, metabolite-only and metabolite + demographic/clinical data models are shown in Supplementary Table 2.

### Models to prognose or predict those who will develop mild COVID-19 symptoms vs. those who will develop severe COVID-19 symptoms

**Figure 2:**
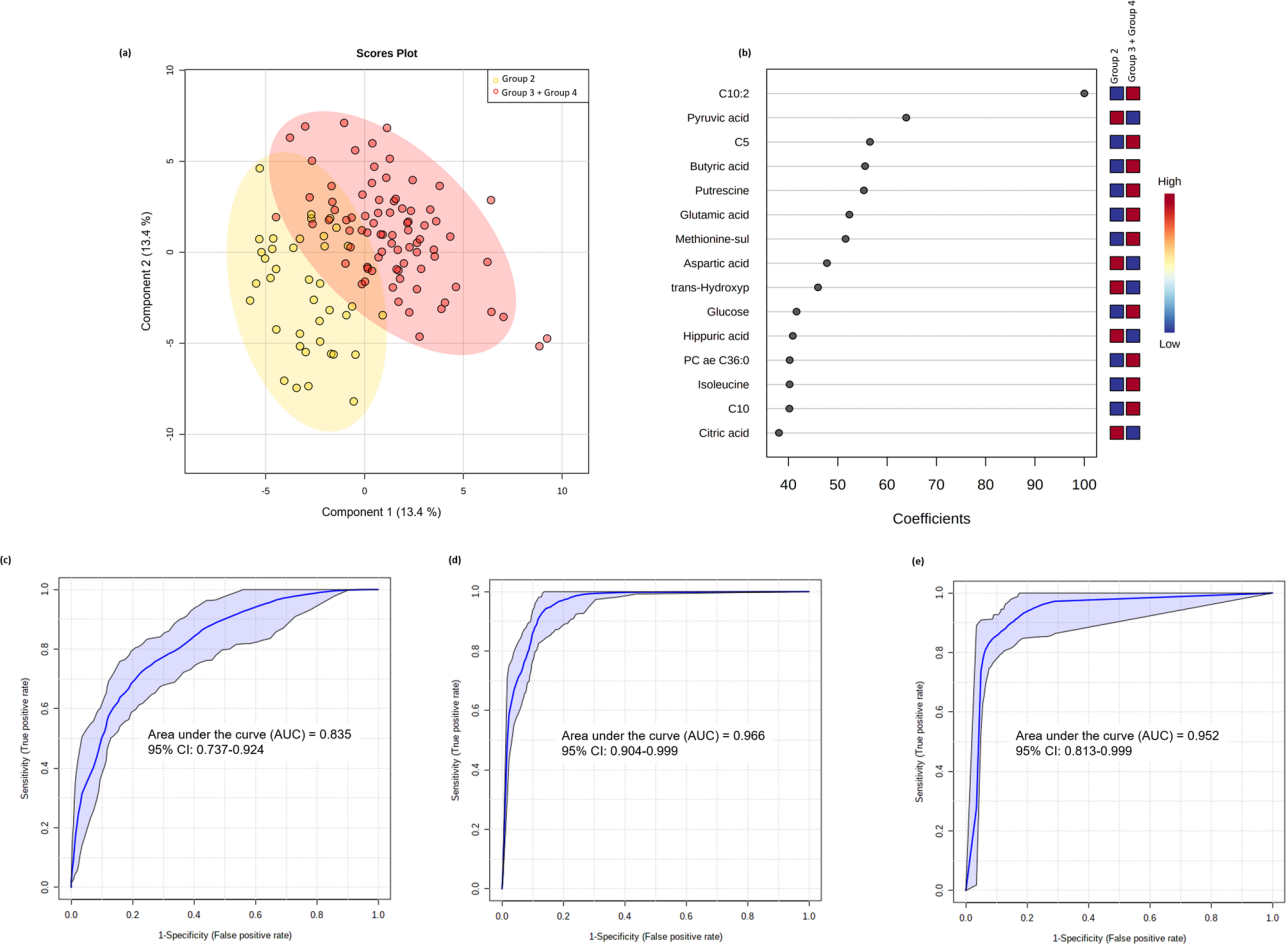
Multivariate analysis from plasma metabolome profile of G2 versus G3 and G4 patients. (**a**) Score scatter plot based on the PLS-DA models to explain the diagnosis (yellow for G2 and red for G3 + G4; (**b**) rank of the different metabolites (the top 15) identified by the PLS-DA according to the VIP coefficient on the x-axis. The most discriminating metabolites are shown in descending order of their coefficient scores. The color boxes indicate whether metabolite concentration is increased (red) or decreased (blue) in G2 vs G3 + G4; (**c**) ROC curve of the demographic/clinical data model; (**d**) ROC curve of the metabolite-only model; (**e**) ROC curve of the metabolite + demographic/clinical data model. The figures were drawn via MetaboAnalyst software v 4.0 (https://www.metaboanalyst.ca/).

In our study, G3 and G4 were hospitalized patients, classified as patients with severe disease based on arterial oxygen tension (PaO_2_)/fractional inspired oxygen (FiO_2_) ratio < 100 at the moment of admission. Figure 2 shows the results of comparisons between patients who developed a mild disease and were not hospitalized (G2) and patients that developed severe disease and were hospitalized (G3 + G4). Multivariate PLS-DA showed a clear separation between both groups (Fig. 2a); C10:2, pyruvic acid, C5 and butyric acid had the highest scores diving the cluster separation (Fig. 2b). The ROC curve with a 95% CI for demographic/clinical data model is shown in Fig. 2c. The AUC, sensitivity and specificity values with 95% CI are shown in Table 2. A logistic regression model was then built with the following equation: logit(P) = log(P/(1 − P)) = 1.089 − 2.36 lymphocytes (%) + 2.226 neutrophils (%) − 0.952 diabetes, where the numeric value of each named metabolite in the equation is the concentration after log transformation and auto-scaling.

The ROC curve with a 95% CI for metabolites only-model is shown in Fig. 2d. The AUC, sensitivity and specificity values with 95% CI are shown in Table 2. A logistic regression model was then built with the following equation: logit(P) = log(P/(1 − P)) = 2.066 + 5.209 C10:2 + 1.948 butyric acid − 2.232 pyruvic acid, where the numeric value of each named metabolite in the equation is the concentration after log transformation and auto-scaling.

When lymphocytes plus neutrophils counts (%) were added to the model (Fig. 2e), the AUC and specificity improved with respect to that of metabolite-only model (Table 2). The equation of the logistic regression model with metabolites and clinical data is: logit(P) = log(P/(1 − P)) = 2.34 + 6.549 C10:2 + 2.516 butyric acid − 2.479 pyruvic acid + 2.166 neutrophils (%) − 2.945 lymphocytes (%).

Additional details for demographic/clinical data, metabolite-only and metabolite + demographic/clinical data are shown in Supplementary Table 3.

### Models to prognose those severe COVID-19 patients who will be intubated

**Figure 3:**
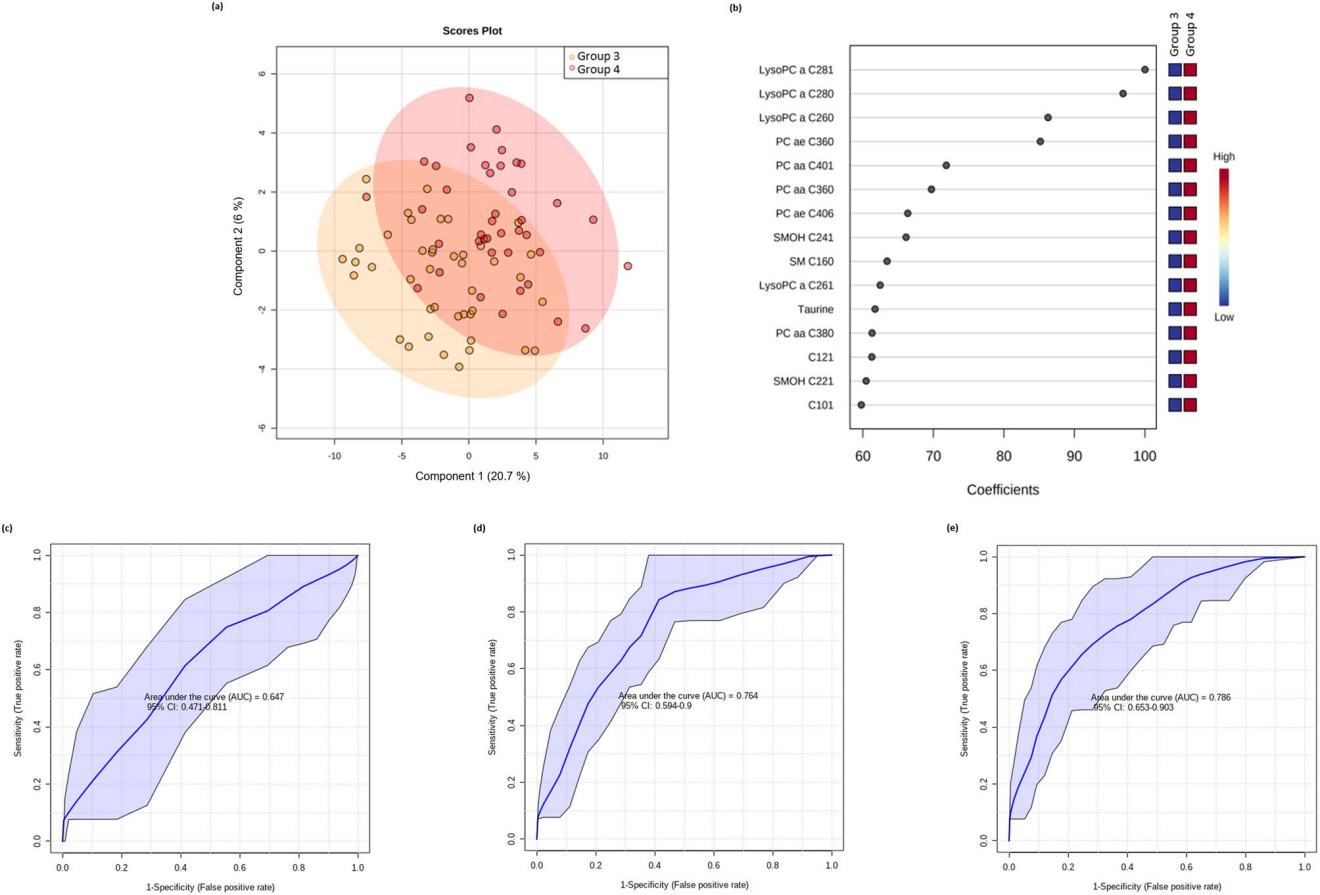
Multivariate analysis from plasma metabolome profile of G3 versus G4 patients. (**a**) Score scatter plot based on the PLS-DA models to explain the diagnosis (orange for G3 and red for G4; (**b**) rank of the different metabolites (the top 15) identified by the PLS-DA according to the VIP coefficient on the x-axis. The most discriminating metabolites are shown in descending order of their coefficient scores. The color boxes indicate whether metabolite concentration is increased (red) or decreased (blue) in G3 vs G4; (**c**) ROC curve of the demographic/clinical data model; (**d**) ROC curve of the metabolite-only model; (**e**) ROC curve of the metabolite + demographic/clinical data model. The figures were drawn via MetaboAnalyst software v 4.0 (https://www.metaboanalyst.ca/).

Forty patients were intubated in our study based on PaO_2_/FiO_2_ and their clinical conditions at the time of admission. However, our data (Table 1) showed close clinical, demographic and metabolic (Fig. 3a) similarities between both study groups, which indicates that the decision criteria for intubation needs to be improved by adding additional metabolic markers. When comparing both groups (G3 and G4), lysoPC a 28:1, lysoPC a 28:0 and lysoPC a 26:0 had the highest scores driving cluster separation (Fig. 3b). The ROC curve with 95% CI for demographic/clinical data is shown in Fig. 3c. The AUC, sensitivity and specificity values with 95% CI are shown in Table 2. A logistic regression model was then built with the following equation: logit(P) = log(P/(1 − P)) = − 0.063 + 0.539 NLR, where the numeric value of each named metabolite in the equation is the concentration after log transformation and auto-scaling.

The ROC curve with 95% CI is shown in Fig. 3d. The AUC, sensitivity and specificity values with 95% CI are shown in Table 2. A logistic regression model was then built with the following equation: logit(P) = log(P/(1 − P)) = − 0.095 + 1.201 lysoPC a C28:0, where the numeric value of each named metabolite in the equation is the concentration after log transformation and auto-scaling.

When hypertension and NLR were added to the model (Fig. 3e), the AUC and specificity improved relative to that of metabolite-only model (Table 2). The equation of the logistic regression model is: logit(P) = log(P/(1 − P)) = − 0.23 + 1.558 lysoPC a C28:0 + 0.831 NLR − 0.768 hypertension.

Additional details for demographic/clinical data, metabolite-only and metabolite + demographic/clinical data are shown in Supplementary Table 4.

**Figure 4:**
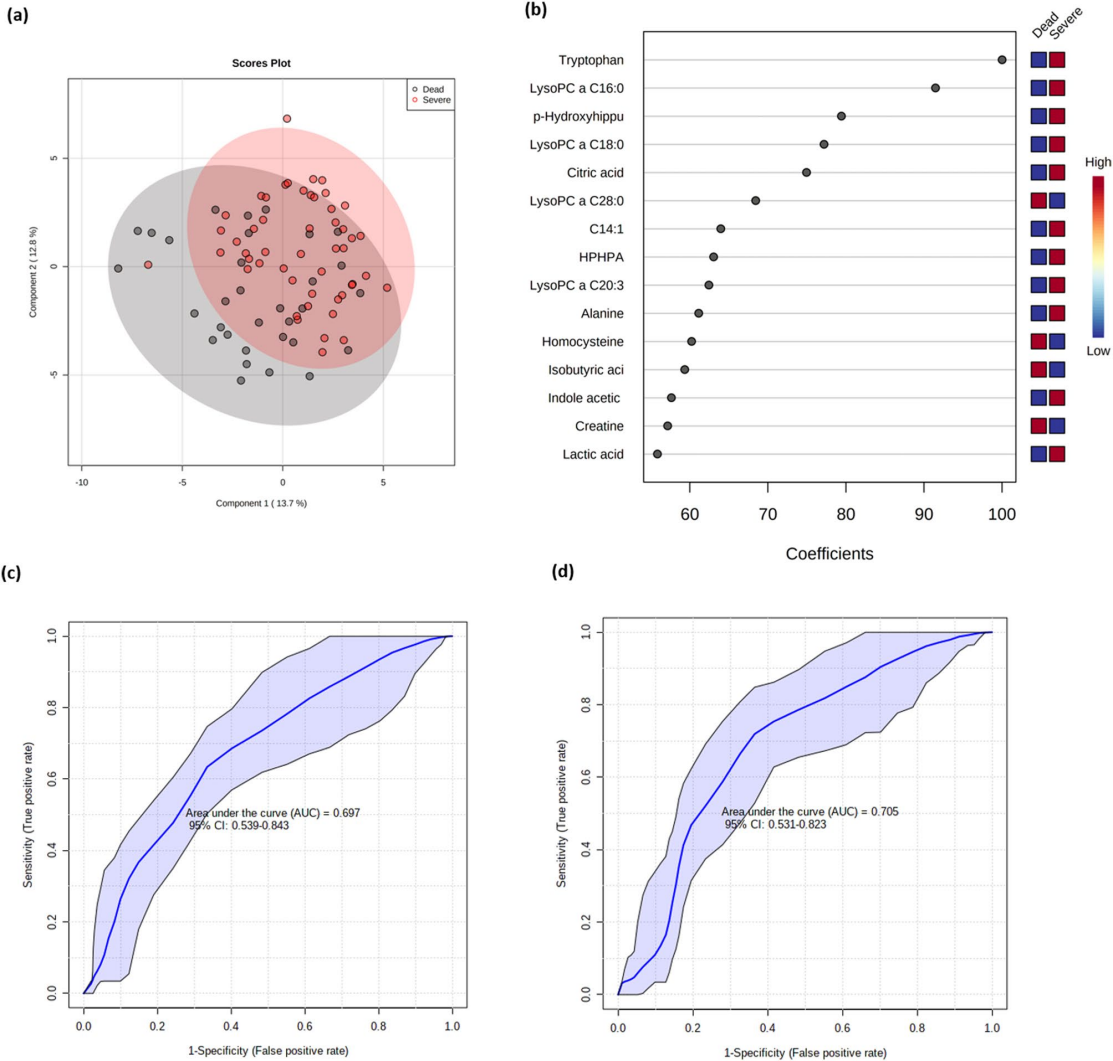
Multivariate analysis from plasma metabolome profile of severe patients versus non-survivors. (**a**) Score scatter plot based on the PLS-DA models to explain the diagnosis (red for severe patients and black for non-survivors, (**b**) rank of the different metabolites (the top 15) identified by the PLS-DA according to the VIP coefficient on the x-axis. The color boxes indicate whether metabolite concentration is increased (red) or decreased (blue) in non-survivors vs severe patients (**c**) ROC curve of the metabolite-only model; (**d**) ROC curve of the metabolite + demographic/clinical data model. The figures were drawn via metaboanalyst software v 4.0 (https://www.metaboanalyst.ca/).

From the 82 patients who developed a severe disease (G3 + G4), 39% died (11 from G3 and 21 from G4). The models based on metabolites-only and metabolites + demographic/clinical data did not differ in this case, showing a low predictive value (Table 2). Figure 4 shows the results of comparisons between patients who developed a severe disease and non-survivors. Multivariate PLS-DA did not show a clear separation between both groups (Fig. 4a); lysoPC a 28:0, lysoPC a 28:1, lysoPC a 26:0 had the highest scores diving the separation (Fig. 4b). The ROC curve for both models with a 95% CI are shown in Fig. 4c,d. It was not possible to build a predictive model of classification based on demographic and clinical data only.

A logistic regression model was then built with the following equation: logit(P) = log(P/(1 − P)) = 0.491 + 0.726 lysoPC a C16:0, where the numeric value of each named metabolite in the equation is the concentration after log transformation and auto-scaling. When age was added to the model (Fig. 4d), the equation of the logistic regression model is: logit(P) = log(P/(1 − P)) = 0.508 + 0.7 lysoPC a C16:0 − 0.451 age. Additional details for metabolite-only and metabolite + demographic/clinical data are shown in Supplementary Table 5.

## Discussion

This study was done to assess the plasma metabolome of COVID-19 patients using targeted, fully quantitative metabolomics methods to produce diagnostic and predictive biomarker panels at different levels of disease severity. Specifically, we wished to develop panels that could: (a) accurately distinguish mild COVID-19 positive from COVID-19 negative patients; (b) prognose or predict those who will develop mild COVID-19 symptoms vs. those who will develop severe COVID-19 symptoms; (c) prognose those severe COVID-19 patients who will need to be intubated and (d) predict those COVID-19 patients with severe disease who will die. Accurate concentrations of 110 metabolites were measured from samples collected immediately after hospital admission and prior to any knowledge of each patient’s COVID-19 status or disease outcome. We also built models adding relevant demographic and clinical data available from the medical records, to improve the predictive value of the metabolite-only models.

Through our analysis we were able to propose a COVID-19 diagnostic panel (panel 1) consisting of three metabolites including the kynurenine/tryptophan ratio, lysoPC a C26:0, and pyruvic acid to discriminate PCR−/controls from PCR+/not hospitalized with a high AUC (0.947 (0.931–0.962)). In addition, another panel (panel 2) consisting of three metabolites, including C10:2, butyric acid, and pyruvic acid could be used to predict which patients would be PCR+/not-hospitalized versus those who would be PCR+/hospitalized and developed a severe disease, with a very high AUC (0.975 (0.968–0.983)). Only lysoPC a C28:0 was used to differentiate between PCR+/hospitalized from PCR+/intubated patients, but with notably lower AUC (0.770 (0.736–0.803)). Adequate metabolic biomarkers to predict which of the hospitalized patients will require mechanical ventilation at a proper time may reduce the risk of mortality.

Efforts to find a useful biomarker panel (panel 4) to predict mortality among those with severe COVID-19 were only modestly successful. In a previous study, Fraser et al.^14^ identified creatinine as the top metabolite for predicting COVID-19-associated mortality on both ICU days 1 and 3, and both creatinine and creatinine/arginine ratio accurately predicted COVID-19-associated death with 100% accuracy (*p *= 0.01)^14^. However, in our study the samples were collected early after hospital admission and due to the cross-sectional design of our study, we did not collect blood samples in a longitudinal manner, that could allow a better outcome prognosis. At the moment of blood sampling, the clinical parameters from the survivors and patients who died, were similar, except for age and lymphocytes count (Supplementary Table 1). This is in line with a recently published longitudinal study, where Sun et al.^20^ found that clinical data resolution was insufficient to clarify critical patients with different outcomes (survivors and non-survivors). In another study, inspection of the global metabolomic profiles of patients that were hospitalized in standard conditions (not in the ICU) did not revealed any major shift that would distinguish the favorable versus unfavorable evolution of COVID-19 neither at baseline nor at day 7^15^.

As showed in Table 2, for each built model, predictive values were lower for demographic/clinical data and higher for metabolites + demographic/clinical data. When demographic and clinical data were added to the four panels (sex and neutrophils counts for the panel 1; lymphocytes and neutrophils counts for the panel 2 and hypertension and diabetes for the panel 3, age for panel 4), the predictive values increased but more so for panels 2 and 3, indicating the influence of factors such as age, sex and comorbidities in contributing to COVID-19 outcomes.

While earlier COVID-19 studies have proposed panels of up to ten metabolites to identify infected patients from healthy individuals^13,21^, and still other studies have proposed individual diagnostic metabolites^12^ or metabolite ratios^14^, the metabolite panels offered here have some advantages. In particular we have identified metabolite-only and metabolite + clinical feature panels that can: (1) reliably distinguish between mildly COVID-19 positive patients and COVID-19 negative patients; and (2) differentiate among three different levels of COVID-19 severity. Furthermore, the panels we have developed include only a few highly predictive metabolites (for the metabolite-only panels) or only a modest number of clinical features (for the metabolite + demographic/clinical data panels). This latter feature means that some of these assays could potentially be adapted into point-of-care settings. For instance, butyric acid, pyruvic acid, kynurenine and tryptophan can be measured with enzyme-linked immunosorbent assays (ELISA) or even simple colorimetric assays. Likewise, MS assays for a small number (3–4) of metabolites can often be done in a matter of minutes at very low cost. Therefore, we believe that if future studies with targeted metabolomics validate and reproduce the diagnostic value of these biomarkers, such panels could eventually be of use in clinical practice.

As noted earlier, our metabolite panel that includes kynurenine/tryptophan ratio, lysoPC a C26:0, and pyruvic acid can discriminate very well (AUC = 0.95) between non-COVID-19 controls (G1) and non-hospitalized COVID-19 patients (G2). The kynurenine pathway is the main route for tryptophan catabolism in the body. It is also a de novo NAD + biosynthetic pathway that is particularly sensitive to the redox environment, and it leads to the production of metabolites with redox capacity. Kynurenine metabolites have been implicated in the physiopathology of many diseases and processes that share common mechanisms. These include the dysregulation of calcium homeostasis, mitochondrial dysfunction, oxidative stress, inflammation and cell death^22^. Indoleamine 2,3-dioxygenase (IDO) activity causes a decrease in tryptophan levels and an accumulation of uncharged tryptophan transfer RNA (Trp-tRNA). Higher levels of Trp-tRNA result in the activation of a protein known as general control non-derepressible 2 (GCN2), a stress-response kinase, which phosphorylates the eukaryotic initiation factor-2 (eIF-2), leading to decreased T-effector cell proliferation. IDO also leads to an increase in kynurenine levels in plasma. Kynurenine binds to the aryl hydrocarbon receptor, causing the differentiation of T-regulatory cells (Tregs) that suppress immune responses and increase IL-6 levels^23^. Significant alterations in the kynurenine pathway have also been reported for COVID-19 patients^11,24^. Thus, this study reinforces the findings that amino acid pathways (especially tryptophan) are significantly dysregulated in COVID-19 infection^10^.

High levels of very long-chain saturated and monounsaturated LysoPCs were also found among COVID-19 patients in this study. In contrast, lysoPC a 16:0 and lysoPC a 18:0 are higher in non-infected controls and patients with mild disease. These lipids were also found higher in survivors from a severe disease than in non-survivors. Decreased plasma LPC levels are associated with unfavorable disease outcomes; for example, plasma LPC levels are decreased in sepsis and correlate inversely with sepsis mortality and in-hospital mortality in pneumonia^25,26^. It has been demonstrated that the acyl chain length and saturation may impact biological activity and function of different LPCs^27^ and this may explain the different patterns found by us in our study. Some authors have suggested that the modulation of host glycerophospholipid metabolism is to support virus replication^28–30^, but most importantly, LPCs have been recognized as important homeostatic mediators involved in inflammation and activation of immune cells^31^. LPCs have been found to act as a strong chemoattractant for monocytes, T cells, as well as natural killer (NK) cells, attracting them to sites of inflammation^31^.

Likewise, a metabolite panel that includes C10:2, butyric acid, and pyruvic acid performed even better (AUC = 0.99) differentiating between non-hospitalized from hospitalized severe COVID-19 patients. As has been noted in many other studies, inflammatory conditions increase CPT-1 activity and cellular stress (i.e. oxidative and hypoxia), while fatty acid oxidation is reduced. In this study, we found that the CPT-1 rate increased among controls compared with intubated COVID-19 patients (Supplementary Fig. 1), suggesting that there is no restriction in the first translocation step of acylcarnitine synthesis. It was also found that the β-oxidation rates for C5 and C10:2 were significantly reduced from group 2 to group 4 (i.e. from mild to critical presentation). Yet, when ratios were calculated for COVID-19 or uninfected controls, with or without metabolic dysregulation (i.e. presence of diabetes mellitus-II, hypertension or obesity), no differences in CPT-1 and β-oxidation ratios were found, suggesting that the differences observed among COVID-19 patients are the result of SARS-CoV-2 infection. Thus, the altered metabolites appear to represent a reprogramming in energy pathways during the illness.

Microbial metabolism dysregulation appears to be implicated in COVID-19 pathogenesis too^32^. In spite of the beneficial action attributed to small chain fatty acids (SCFAs)^33^, there is also evidence of the potential role of propionic and butyric acids in the etiology of irritable bowel syndrome (IBS). In particular, increased serum levels of these metabolites have been found in patients with this condition and it is notable that diarrhea is one of the symptoms that is common to both COVID and IBS^34^. In addition, high levels of butyric acid have been thought to promote viral replication and interfere with the interferon response^35^. Therefore, the dysregulation of gut microbiota, expressed by various gastrointestinal symptoms and an altered profile of microbial metabolites, underscores the potential relevance of dietary interventions to prevent the progression of COVID-19 severity. It is possible that modifying fiber and g fatty components to the diet could modify plasma levels of SCFAs in COVID-19 patients.

In this study, pyruvic acid was found differentially expressed across most groups (increased: Group 2 vs. Group 1; decreased: Group 3 vs. Group 2). Pyruvate is a critical metabolite in glycolysis, and it is likely that viral infection or the body’s response to viral infection causes a shift from oxidative phosphorylation to aerobic glycolysis to accelerate the synthesis of nucleotides, amino acids, and fatty acids needed to support the body’s immune response (to SARS-CoV2). Evidence suggests that cells adapt their metabolism upon viral infection to become glycolytic. In particular, infections appear to trigger mitochondrial reactive oxygen species production, which leads to a stabilization of hypoxia-inducible factor-1α (HIF-1α), inducing glycolysis^36^ and the subsequent increase of pyruvic acid during the cell’s response to viral replication. Actually, high levels of pyruvic acid, previously attributed to a dysregulation of the hepatic central carbon metabolism, have been noted in COVID-19 patients previously^37^. On the other hand, the lack of oxygen seen in patients with severe COVID-19 symptoms will tend to activate the tricarboxylic acid cycle (TCA). As a result, pyruvate will be converted to lactate due to aerobic glycolysis, which is also known as the Warburg effect^38^. Thus, a reduction in TCA metabolism would lead to an anti-oxidant imbalance and inflammatory damage^10^, as seen by the increased levels of polyamines and methionine sulfoxide found among hospitalized patients in this study.

We believe that the panels proposed by us in the present work have excellent predictive values compared with values reported for serological tests [(AUC (IgM): 0.81; AUC (IgG): 0.93; AUC (IgM + IgG): 0.98)]^39^; and the RT-PCR test (AUC: 0.83)^40^. Studies aimed at evaluating the accuracy of laboratory parameters (neutrophil count, lactate dehydrogenase, aspartate aminotransferase, alanine aminotransferase and urea) in predicting COVID-19 cases with positive RT-PCR for COVID-19 have demonstrated an AUC of just 0.8^41^. With the combined panels that we propose, we may distinguish/predict mild infections from non-infected individuals, which ones will be severe or even intubated and which ones will be mild, as well as which ones will live and which ones will die, which is only possible by assessing metabolomics and/or clinical-chemistry analysis. Based on the lipid and energy metabolism alterations described in the present work, we are also providing insights about possible molecular targets to inhibit virus replication and proliferation, such as some currently under study (fatty acid synthase inhibitors, PI3 kinase inhibitors, AMPK activators, etc.).

## Limitations of the study

As with any study associated with COVID-19 and other newly emerging diseases, there are a number of limitations that ought to be mentioned. One limitation is the categorization we chose to use as a proxy for disease severity could have led to misclassification bias, particularly for patients in group 3 (PCR+/hospitalized) and group 4 (PCR+/intubated), and to a lesser extent from those in group 2 (PCR+/not hospitalized) compared to group 3 (PCR+/hospitalized). This arose due to the lack of hospital beds available, especially ICU beds, forcing physicians at our hospital to keep critically ill patients needing intubation in the regular hospitalization area instead of the ICU, or to provide ambulatory care to patients that required hospitalization with an oxygen mask. Moreover, due to the lack of rigorous tools to classify disease severity, protocols for classifying patients upon their admission to the ICU need to be improved with more accurate measurements, such as those that we and others propose. In fact, this bias could have been the reason for the incomplete identification of significant metabolites distinguishing between groups 3 and 4. Another limitation with this study lies in the partial selection of metabolites based on VIP and LASSO scores that could have resulted in less accurate predictive models. We deliberately limited the number of metabolites selected for regression analyses to develop metabolite panels that were more amenable for clinical application and more rapid testing. A third limitation concerns the cross-sectional exploratory nature of the study design. This design prevented a longitudinal metabolite assessment and so further prospective observational designs, such a cohort with repetitive measures would be needed to follow-up on the measured metabolite concentrations throughout the progression of the infection and to better distinguish those with severe COVID-19 who will recover and those who will die. Finally, it is important to note that nutrition habits and or/medications taken prior to the blood sample collection could have affected some metabolite concentrations. However, this issue appears to have had a limited impact, as only 25% of the surveyed patients reported having taken symptomatic treatment (antipyretics and analgesics) prior to blood sampling.

## Methods

### Patients enrollment and sample collection

A sample of 161 symptomatic individuals aged 35–70 years were included for metabolite analyses. These patients were taken from a set of 1058 patients who were RT-qPCR-tested for SARS-CoV-2 between March 15 and November 1, 2020 at the Zacatecas General Hospital’s Respiratory Triage Unit of by the Mexican Institute of Social Security (IMSS). This is a public facility with ≈ 200 hospital beds located in Zacatecas, the capital city of the state of Zacatecas in central Mexico. Screened individuals were categorized into four mutually exclusive groups: Group 1: PCR−, controls (n = 39); Group 2: PCR+, not hospitalized (n = 40); Group 3: PCR+, hospitalized with or without oxygen mask (n = 42); and Group 4: PCR+, intubated (n = 40). Blood specimens for plasma analyses were collected within two days after admission on average. Baseline information such as age, gender, comorbidities, clinical and laboratory data, and outcome (survival or non-survival) was obtained from the electronic medical records of each patient and stored by a password-protected database and provided in Table 1. The study was performed in accordance with the Declaration of Helsinki. It was also revised and approved by the Ethics Committee of the Comité Nacional de Investigación Científica del Instituto Mexicano de Seguridad Social, with the registration number R-2020-785-068. Informed consent was obtained from all participants. All patients included in this study were informed in writing regarding the collection of their samples for research aims and given the right to refuse such uses.

### Metabolomics profile of plasma samples

Targeted quantitative metabolomics was used to identify and determine the concentration of 143 different endogenous metabolites. Amino acids, biogenic amines and derivatives, and organic acids were analyzed using a reverse-phase liquid chromatography-mass spectrometry (LC–MS)/MS custom assay. Glycerophospholipids, acylcarnitines, sphingomyelins, and glucose were measured by direct injection (DI). Mass spectrometric analyses were performed on an ABSciex 4000 Qtrap tandem MS instrument (Applied Biosystems/MDS Analytical Technologies, Foster City, CA, USA) equipped with an Agilent 1260 series UHPLC system (Agilent Technologies, Palo Alto, CA). The custom assay contained a 96-deep-well plate with a filter plate attached with sealing tape; reagents and solvents were used to prepare the plate assay. The first 14 wells were used for one blank, three zero samples, seven standards, and three quality control samples. Details of the assay have been published previously^19^.

### Sample preparation

For organic acid analyses, 150 µL of ice-cold methanol and 10 µL of isotope-labeled internal standard mixture were added to 50 µL of plasma sample for overnight protein precipitation at − 20 °C, followed by centrifugation at 13,000×*g* for 20 min. A total of 50 µL of supernatant was loaded into the center of a 96-deep-well plate, followed by the addition of a 3-nitrophenylhydrazine reagent. After incubation for 2 h, butylated hydroxytoluene stabilizer (2 mg/mL) and water were added before the LC–MS injection.

For amino acids and biogenic amines and derivatives, glycerophospholipids, acylcarnitines, and sphingomyelins, samples were thawed on ice and subsequently vortexed and centrifuged at 13,000×*g*; 10 µL of each sample was then loaded onto the center of the filter on the upper 96-well plate and dried in a stream of nitrogen. Subsequently, phenyl-isothiocyanate was added for derivatization. After incubation, the filter spots were dried again using an evaporator. Metabolite extraction was then achieved by adding 300 µL of extraction solvent. Extracts were obtained by centrifugation into the lower 96-deep-well plate, followed by a dilution step with MS running solvent (0.2% formic acid in water, 0.2% formic acid in acetonitrile).

### LC–MS/MS method

An Agilent reversed-phase Zorbax Eclipse XDB C18 column (3.0 mm × 100 mm, 3.5 μm particle size, 80 Å pore size) with a Phenomenex (Torrance, CA, USA) SecurityGuard C18 pre-column (4.0 mm × 3.0 mm) was used. LC parameters used were as follows: mobile phase A was 0.2% (v/v) formic acid in water, and mobile phase B was 0.2% (v/v) formic acid in acetonitrile. The gradient profile was: t = 0 min, 0% B; t = 0.5 min, 0% B; t = 5.5 min, 95% B; t=6.5 min, 95% B; t = 7.0 min, 0% B; and t = 9.5 min, 0% B. The column oven was set at 50 °C. The flow rate was 500 μL/min, and the sample injection volume was 10 μL.

For the analysis of organic acids, the mobile phases used were (A) 0.01% (v/v) formic acid in water, and (B) 0.01% (v/v) formic acid in methanol. The gradient profile was as follows: t = 0 min, 30% B; t = 2.0 min, 50% B; t = 12.5 min, 95% B; t = 12.51 min, 100% B; t = 13.5 min, 100% B; t = 13.6 min, 30% B and finally maintained at 30% B for 4.4 min. The column oven was set to 40 °C. The flow rate was 300 μL/min, and the sample injection volume was 10 μL.

### DI-MS/MS method

The LC autosampler was connected directly to the MS ion source by red PEEK tubing. The mobile phase was prepared by mixing 60 μL of formic acid, 10 mL of water, and 290 mL of methanol. The flow rate was programmed as follows: t = 0 min, 30 μL/min; t = 1.6 min, 30 μL/min; t = 2.4 min; 200 μL/min; t = 2.8 min, 200 μL/min; and t = 3.0 min, 30 μL/min. The sample injection volume was 20 μL.

### Quantification

To quantify organic acids, amino acids, and biogenic amines and derivatives, an individual seven-point calibration curve was generated for each analyte. Ratios for each analyte’s signal intensity to its corresponding isotope-labelled internal standard were plotted against the specific known concentrations using quadratic regression with a 1/x^2^ weighting.

Lipids, acylcarnitines, and glucose were analyzed semi-quantitatively. A single point calibration of a representative analyte was built using the same group of compounds that share the same core structure assuming a linear regression through zero.

All metabolite analyses were done using Analyst 1.6.2 and MultiQuant 3.0.3.

### Statistical analysis

Frequencies and proportions stratified by study group were used to describe nominal variables. Since continuous data was not normally distributed, medians with quartiles 1 (Q1) and 3 (Q3) were used as central and dispersion measures stratifying by surveyed group.

Metabolites with > 50% of missing values were removed from further analysis (n = 33). Half of the minimum concentration value was imputed in those with < 50% of missing values. Metabolites were log-transformed and auto-scaled. Principal component analysis (PCA) and two-dimension partial least squares discriminant analysis (2-D PLS-DA) scores plots were used to compare plasma metabolite data across and between study groups; 2000-fold permutation tests were used to minimize the possibility that the observed separation of the PLS-DA was due to chance. Coefficient scores and least absolute shrinkage and selection operator (LASSO) algorithm were used to identify the most discriminating metabolites for group comparisons. Metabolite data analyses was done using MetaboAnalyst^42^.

The metabolites with the highest score coefficient and LASSO scores were used to create these metabolite panels for COVID-19 status or outcomes using multivariate logistic regression (demographic/clinical data and metabolites-only models). Additionally, models were adjusted for relevant potential confounders such as sex, age, relevant comorbidities (i.e. DM-II, HTN, and obesity), and clinical laboratory data, but only statistically significant variables (p < 0.05) remained in the final models (metabolites + demographic/clinical data models). Logistic regression analysis was performed with the generalized log-transformed and auto scaled data. The stepwise variable selection was also utilized for optimizing all the model components. Furthermore, a k-fold cross-validation (CV) technique was used to ensure that the logistic regression models were robust. In k-fold CV, the entire sample data is randomly divided into k equal sized subsets. Of the k subsets, only one subset is used as the validation data for testing the model, and the remaining (k − 1) subsets are used as training set to generate the model. This results in predictive biomarker predictive models that are both robust and optimal. To determine the performance of each generated model, the area under the receiver operating characteristics curve (AUROC or AUC) was calculated with sensitivity and specificity. Balanced sub-sampling-based Monte Carlo cross validation (MCCV) was used to generate the ROC curves. The 95 % CI for the AUC curves were also calculated. All these analyses were performed using the MetaboAnalyst software.

### Ethics declarations

The study was performed in accordance with the Declaration of Helsinki. It was also revised and approved by the Ethics Committee of the Comité Nacional de Investigación Científica del Instituto Mexicano de Seguridad Social, with the registration number R-2020-785-068. Informed consent was obtained from all participants. All patients included in this study were informed in writing regarding the collection of their samples for research aims and given the right to refuse such uses.

## Supplementary Information

Supplementary Information.

## Data Availability

All the clinical and metabolomic data produced in this work are available from the Mendeley Data Repository: 10.17632/7fnt3nfhdv.1.
