# Author Correction: Targeted metabolomics identifies high performing diagnostic and prognostic biomarkers for COVID-19

**DOI:** 10.1038/s41598-022-05978-2

**Published:** 2022-01-25

**Authors:** Yamilé López-Hernández, Joel Monárrez-Espino, Ana-Sofía Herrera-van Oostdam, Julio Enrique Castañeda Delgado, Lun Zhang, Jiamin Zheng, Juan José Oropeza Valdez, Rupasri Mandal, Fátima de Lourdes Ochoa González, Juan Carlos Borrego Moreno, Flor M. Trejo-Medinilla, Jesús Adrián López, José Antonio Enciso Moreno, David S. Wishart

**Affiliations:** 1 Cátedras-CONACyT, Consejo Nacional de Ciencia y Tecnologia, 03940 Mexico, Mexico; 2 Autonomous University of Zacatecas, 98000 Zacatecas, Mexico; 3 Christus Muguerza Hospital Chihuahua-University of Monterrey, 31000 Chihuahua, Mexico; 4 Faculty of Medicine, Autonomous University of San Luis Potosí, 78210 San Luis Potosi, Mexico; 5 Unidad de Investigación Biomédica de Zacatecas, Instituto Mexicano del Seguro Social, 98000 Zacatecas, Mexico; 6 The Metabolomics Innovation Center, University of Alberta, Edmonton, AB T6G1C9 Canada; 7 Doctorado en Ciencias Básicas, Universidad Autónoma de Zacatecas, Zacatecas, México; 8 Departmento de Epidemiología, Hospital General de Zona #1 “Emilio Varela Luján”, Instituto Mexicano del Seguro Social, 98000 Zacatecas, Mexico; 9 MicroRNAs Laboratory, Academic Unit for Biological Sciences, Autonomous University of Zacatecas, 98000 Zacatecas, Mexico

Correction to: *Scientific Reports* 10.1038/s41598-021-94171-y, published online 19 July 2021

The original version of this Article contained an error in Figure 1b, where the labels of ‘Group 1’ and ‘Group 2’ were mistakenly interchanged. The label for ‘Group 1’ now reads ‘Group 2’ and the label for ‘Group 2’ now reads ‘Group 1’. The error does not alter the results and conclusions.

**Figure 1:**
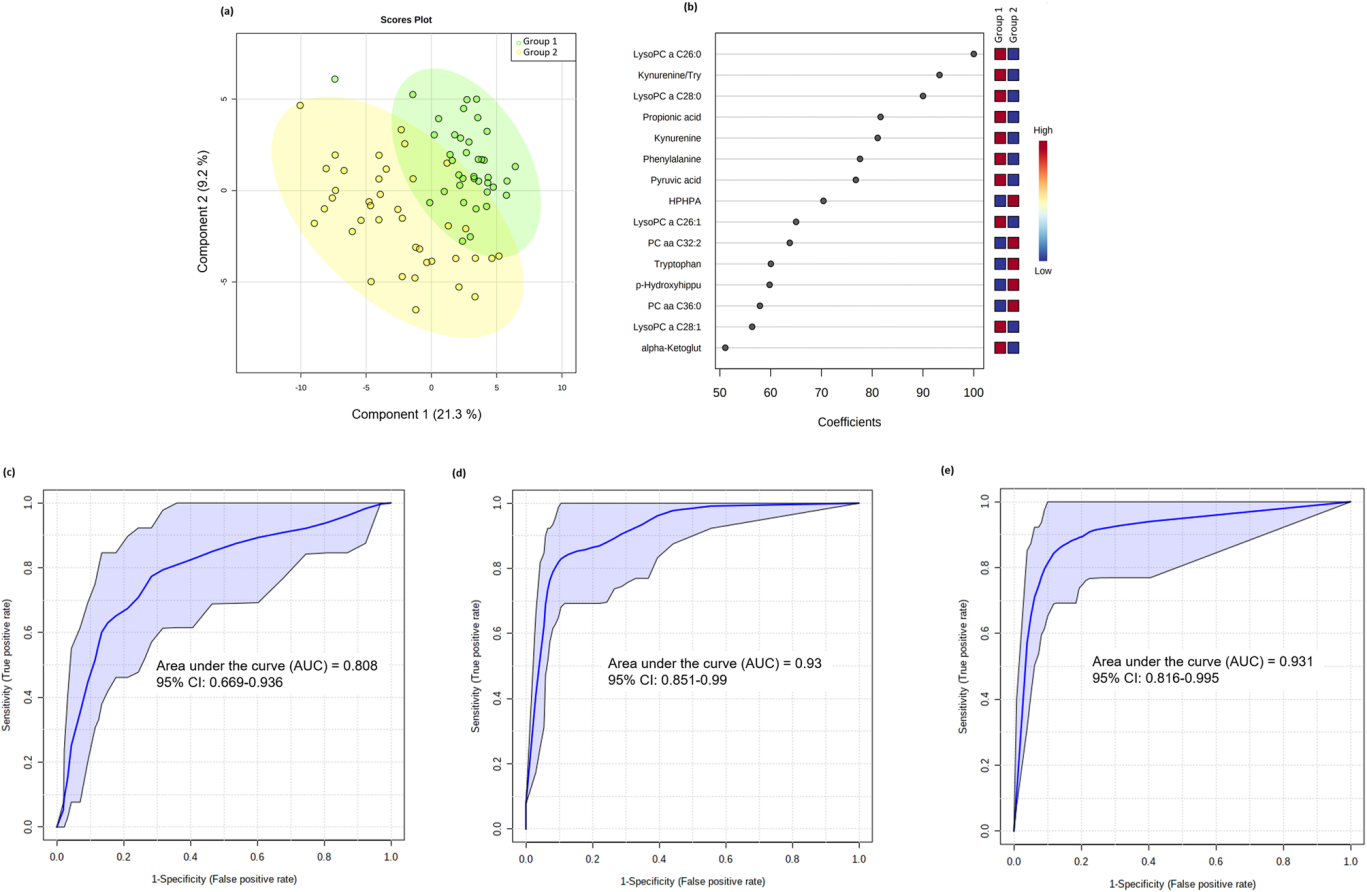
Multivariate analysis from plasma metabolome profile of G1 versus G2 patients. (**a**) Score scatter plot based on the PLS-DA models to explain the diagnosis (green for G1 and yellow for G2; (**b**) rank of the different metabolites (the top 15) identified by the PLS-DA according to the VIP coefficient on the x-axis. The most discriminating metabolites are shown in descending order of their coefficient scores. The color boxes indicate whether metabolite concentration is increased (red) or decreased (blue) in G1 vs G2; (**c**) ROC curve of the demographic/clinical data model; (**d**) ROC curve of the metabolite-only model; (**e**) ROC curve of the metabolite + demographic/clinical data model. The figures were drawn via MetaboAnalyst software v 4.0 (https://www.metaboanalyst.ca/).

The original Figure 1 and accompanying legend appears below.

The original Article has been corrected.

