# Supplementary material for: Targeted metabolomics identifies high performing diagnostic and prognostic biomarkers for COVID-19
Source: Sci Rep. 2021 Jul 19;11:14732. doi: 10.1038/s41598-021-94171-y (PMC8290000; doi:10.1038/s41598-021-94171-y)
Supplement: Supplementary file 1 — Supplementary Information. [file 41598_2021_94171_MOESM1_ESM.docx]

**Targeted metabolomics identifies high performing diagnostic and prognostic biomarkers for COVID-19**

Yamilé López-Hernández,^1,2*^ Joel Monárrez-Espino,^3*^ Ana-Sofía Herrera-van Oostdam,^4^ Julio Enrique Castañeda Delgado,^1,5^ Lun Zhang,^6^ Jiamin Zheng,^6^ Juan José Oropeza Valdéz,^5^ Rupasri Mandal,^6^ Fátima Ochoa González,^5,7^ Juan Carlos Borrego Moreno,^8^ Flor M. Trejo-Medinilla,^2,7^ Jesus Adrian Lopez,^9^ José Antonio Enciso Moreno,^5^ David S. Wishart^6^

^1^ Cátedras-CONACyT, Consejo Nacional de Ciencia y Tecnologia, México 03940, México

^2^ Autonomous University of Zacatecas, Zacatecas 98000, Mexico

^3^ Christus Muguerza Hospital Chihuahua - University of Monterrey, Chihuahua 31000, Mexico

^4^ Faculty of Medicine, Autonomous University of San Luis Potosí, San Luis Potosi 78210, Mexico

^5^ Unidad de Investigación Biomédica de Zacatecas, Instituto Mexicano del Seguro Social, Zacatecas 98000 México.

^6^ The Metabolomics Innovation Center, University of Alberta, Edmonton, AB T6G1C9, Canada, Canada

^7^Doctorado en Ciencias Básicas, Universidad Autónoma de Zacatecas, Zacatecas, México.

^8^Departmento de Epidemiología, Hospital General de Zona #1 “Emilio Varela Luján”, Instituto Mexicano del Seguro Social, Zacatecas 98000, México

^9^MicroRNAs Laboratory, Academic Unit for Biological Sciences, Autonomous University of Zacatecas, Zacatecas 98000, Mexico

**Corresponding authors**

* ylopezher@conacyt.mx; jmonarrez@hotmail.com

**Supplementary Figures**

**Supplementary Figure 1.** CPT-1 and B-oxidation rate in COVID-19 patients and non-COVID-19 subjects

**Supplementary Tables**

**Supplementary Table 1.** Baseline clinical characteristics of survivors and non-survivors from group 3 and group 4

**Supplementary Table 2.** Logistics regression based optimal models for G1 and G2

**Supplementary Table 3.** Logistics regression based optimal models for G2 and G3+G4

**Supplementary Table 4.** Logistics regression based optimal models for G3 and G4

**Supplementary Table 5.** Logistics regression based optimal models for non survivors and severe patients

**
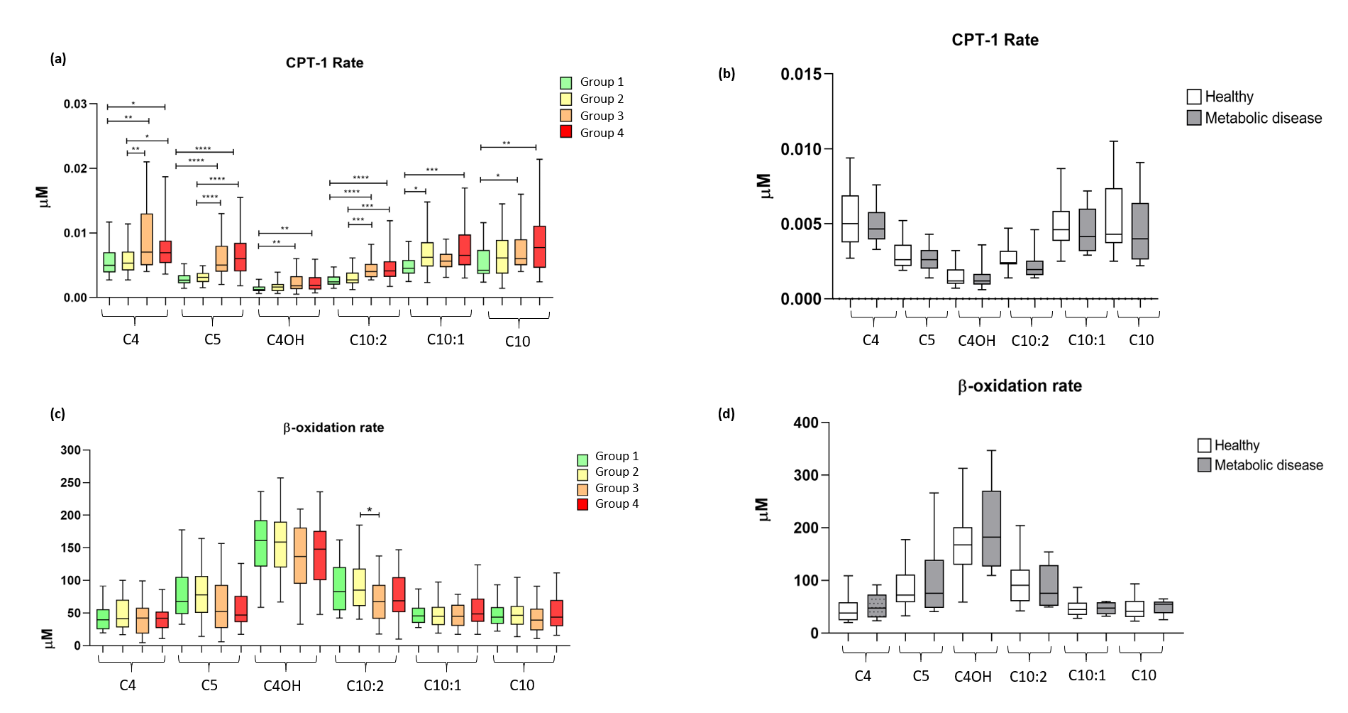
**

**Supplementary Table 1**. Baseline clinical characteristics of survivors and non-survivors from group 3 and group 4

| Variables | Severe-survivors (N = 50) | Non-survivors (N = 32) | p value |
| --- | --- | --- | --- |
| Male sex, n (%) | 32 (64.0) | 17 (53.1) | 0.4 |
| Age, median years (mean ±S D) | 52.8±9.6 | 57.4±9.8 | **0.04** |
| Comorbidities, n (%) |  |  |  |
| Diabetes | 17 (34.0) | 12 (37.5) | 0.8 |
| Obesity | 10 (20.0) | 13 (38.2) | 0.08 |
| Hypertension | 20 (40.0) | 15 (46.9) | 0.6 |
| Lab data, median (Q1-Q3) |  |  |  |
| Leukocytes (×10^3^) | 9.3 (6.6-11.6) | 9.1 (7.6-9.9) | 0.5 |
| Neutrophils (%) | 82.7 (76.0-88.6) | 83.6 (80.2-90.0) | 0.2 |
| Lymphocytes (%) | 12.6 (7.7-16.2) | 10.1 (6.0-11.9) | **0.03** |
| Neutrophils- Lymphocytes Radio (NLR) | 8.7 (5.0-10.0) | 9.7 (7.0-11.0) | 0.09 |
| Monocytes (%) | 4.3 (2.9-5.3) | 4.6 (2.6-5.4) | 0.9 |
| Glucose (mg/dl) | 132.0 (108.6-205.6) | 154.4 (113.5-243.6) | 0.4 |

**Supplementary Table 2**. Logistics regression based optimal models for G1 and G2

| **Demographic/clinical data only** | **Estimate** | **Std. Error** | **z value** | **Pr(>\|z\|)** | **Odds** |
| --- | --- | --- | --- | --- | --- |
| **(Intercept)** | -0.098 | 0.277 | -0.354 | 0.724 | - |
| age | -1.22 | 0.307 | -3.973 | < 0.001 | 0.3 |
| lymphocytes (%) | 0.722 | 0.319 | 2.26 | 0.024 | 2.06 |
| **Logistic Regression Model with Selected Compounds:**  logit(P) = log(P / (1 - P)) = -0.098 - 1.22 age + 0.722 lymphocytes (%)  The optimal cut-off point for the above equation is 0.41. | | | | | |
| **Metabolites only** | **Estimate** | **Std. Error** | **z value** | **Pr(>\|z\|)** | **Odds** |
| **(Intercept)** | -0.444 | 0.409 | -1.086 | 0.278 | - |
| kynurenine/tryptophan | -2.599 | 0.596 | -4.358 | < 0.001 | 0.07 |
| lysoPC a C26:0 | -1.483 | 0.504 | -2.941 | 0.003 | 0.23 |
| pyruvic acid | -1.142 | 0.507 | -2.252 | 0.024 | 0.32 |
| **Logistic Regression Model with Selected Compounds:**  logit(P) = log(P / (1 - P)) = -0.444 - 2.599 kynurenine/tryptophan - 1.483 lysoPC a C26:0 -1.142 pyruvic acid  The optimal cut-off point for the above equation is 0.5. | | | | | |
| **Metabolites and demographic/clinical data** | **Estimate** | **Std. Error** | **z value** | **Pr(>\|z\|)** | **Odds** |
| **(Intercept)** | -0.874 | 0.559 | -1.565 | 0.118 | - |
| kynurenine/tryptophan | -3.659 | 0.918 | -3.987 | < 0.001 | 0.03 |
| lysoPC a C26:0 | -2.168 | 0.719 | -3.013 | 0.003 | 0.11 |
| pyruvic acid | -1.703 | 0.717 | -2.375 | 0.018 | 0.18 |
| sex | 1.05 | 0.518 | 2.028 | 0.043 | 2.86 |
| neutrophils (%) | 1.179 | 0.552 | 2.136 | 0.033 | 3.25 |
| **Logistic Regression Model with Selected Compounds:**  logit(P) = log(P / (1 - P)) = -0.874 - 3.659 kynurenine/tryptophan - 2.168 lysoPC a C26:0 - 1.703 pyruvic acid + 1.05 sex + 1.179 neutrophils (%)  The optimal cut-off point for the above equation is 0.47. | | | | | |

G1: PCR-/controls, G2: PCR+/not hospitalized

**Supplementary Table 3.** Logistics regression based optimal models for G2 and G3+G4

| **Demographic/clinical data only** | **Estimate** | | **Std. Error** | | **z value** | | **Pr(>\|z\|)** | | **Odds** | |
| --- | --- | --- | --- | --- | --- | --- | --- | --- | --- | --- |
| **(Intercept)** | 1.089 | | 0.269 | | 4.046 | | < 0.001 | | - | |
| lymphocytes (%) | -2.36 | | 0.482 | | -4.894 | | < 0.001 | | 0.09 | |
| neutrophils (%) | 2.226 | | 0.491 | | 4.536 | | < 0.001 | | 9.26 | |
| diabetes | -0.952 | | 0.305 | | -3.117 | | 0.002 | | 0.39 | |
| **Logistic Regression Model with Selected Compounds:**  logit(P) = log(P / (1 - P)) = 1.089 - 2.36 lymphocytes (%) + 2.226 neutrophils (%) - 0.952 diabetes  The optimal cut-off point for the above equation is 0.69. | | | | | | | | | | |
| **Metabolites only** | | **Estimate** | | **Std. Error** | | **z value** | | **Pr(>\|z\|)** | | **Odds** |
| **(Intercept)** | | 2.066 | | 0.518 | | 3.984 | | < 0.001 | | - |
| C10:2 | | 5.209 | | 1.165 | | 4.47 | | < 0.001 | | 182.87 |
| butyric acid | | 1.948 | | 0.617 | | 3.159 | | 0.002 | | 7.02 |
| pyruvic acid | | -2.232 | | 0.679 | | -3.288 | | 0.001 | | 0.11 |
| **Logistic Regression Model with Selected Compounds:** logit(P) = log(P / (1 - P)) = 2.066 + 5.209 C10:2 + 1.948 butyric acid - 2.232 pyruvic acid  The optimal cut-off point for the above equation is 0.48 | | | | | | | | | | |
| **Metabolites and demographic/clinical data** | | **Estimate** | | **Std. Error** | | **z value** | | **Pr(>\|z\|)** | | **Odds** |
| **(Intercept)** | | 2.34 | | 0.708 | | 3.304 | | 0.001 | | - |
| C10:2 | | 6.549 | | 2.256 | | 2.903 | | 0.004 | | 698.76 |
| butyric acid | | 2.516 | | 1.156 | | 2.176 | | 0.03 | | 12.38 |
| pyruvic acid | | -2.479 | | 0.964 | | -2.571 | | 0.01 | | 0.08 |
| neutrophils (%) | | 2.166 | | 0.927 | | 2.338 | | 0.019 | | 8.73 |
| lymphocytes (%) | | -2.945 | | 1.096 | | -2.687 | | 0.007 | | 0.05 |
| **Logistic Regression Model with Selected Compounds:**  logit(P) = log(P / (1 - P)) = 2.34 + 6.549 C10:2 + 2.516 butyric acid - 2.479 pyruvic acid + 2.166 neutrophils (%) - 2.945 lymphocytes (%)  The optimal cut-off point for the above equation is 0.82. | | | | | | | | | | |

G2: PCR+/not hospitalized, G3: PCR+/hospitalized, and G4: PCR+/intubated.

**Supplementary Table 4.** Logistics regression based optimal models for G3 and G4

| **Demographic/clinical data only** | **Estimate** | | **Std. Error** | | **z value** | | **Pr(>\|z\|)** | | **Odds** | |
| --- | --- | --- | --- | --- | --- | --- | --- | --- | --- | --- |
| **(Intercept)** | -0.063 | | 0.229 | | 0.229 | | 0.783 | | - | |
| NLR | 0.539 | | 0.246 | | 0.246 | | 0.028 | | 1.71 | |
| **Logistic Regression Model with Selected Compounds:**  logit(P) = log(P / (1 - P)) = -0.063 + 0.539 NLR  The optimal cut-off point for the above equation is 0.52. | | | | | | | | | | |
| **Metabolites only** | | **Estimate** | | **Std. Error** | | **z value** | | **Pr(>\|z\|)** | | **Odds** |
| **(Intercept)** | | -0.095 | | 0.252 | | -0.376 | | 0.707 | | - |
| lysoPC a C28:0 | | 1.201 | | 0.312 | | 3.847 | | < 0.001 | | 3.32 |
| **Logistic Regression Model with Selected Compounds:**  logit(P) = log(P / (1 - P)) = -0.095 + 1.201 lysoPC a C28:0  The optimal cut-off point for the above equation is 0.44. | | | | | | | | | | |
| **Metabolites and demographic/clinical data** | | **Estimate** | | **Std. Error** | | **z value** | | **Pr(>\|z\|)** | | **Odds** |
| **(Intercept)** | | -0.23 | | 0.282 | | -0.814 | | 0.415 | | - |
| lysoPC a C28:0 | | 1.558 | | 0.393 | | 3.965 | | < 0.001 | | 4.75 |
| NLR | | 0.831 | | 0.331 | | 2.508 | | 0.012 | | 2.29 |
| hypertension | | -0.768 | | 0.317 | | -2.422 | | 0.015 | | 0.46 |
| **Logistic Regression Model with Selected Compounds:**  logit(P) = log(P / (1 - P)) = -0.23 + 1.558 lysoPC a C28:0 + 0.831 NLR - 0.768 hypertension  The optimal cut-off point for the above equation is 0.47. | | | | | | | | | | |

G3: PCR+/hospitalized, and G4: PCR+/intubated.

**Supplementary Table 5.** Logistics regression based optimal models for non survivors and severe patients

| **Metabolites only** | **Estimate** | **Std. Error** | **z value** | **Pr(>\|z\|)** | **Odds** |
| --- | --- | --- | --- | --- | --- |
| **(Intercept)** | 0.491 | 0.241 | 2.038 | 0.042 | - |
| lysoPC a C16:0 | 0.726 | 0.263 | 2.763 | 0.006 | 2.07 |
| **Logistic Regression Model with Selected Compounds:**  logit(P) = log(P / (1 - P)) = 0.491 + 0.726 lysoPC a C16:0  The optimal cut-off point for the above equation is 0.61. | | | | | |
| **Metabolites and demographic/clinical data** | **Estimate** | **Std. Error** | **z value** | **Pr(>\|z\|)** | **Odds** |
| **(Intercept)** | 0.508 | 0.246 | 2.061 | 0.039 | - |
| lysoPC a C16:0 | 0.7 | 0.267 | 2.617 | 0.009 | 2.01 |
| age | -0.451 | 0.268 | -1.681 | 0.093 | 0.64 |
| \| **Logistic Regression Model with Selected Compounds:**  logit(P) = log(P / (1 - P)) = 0.508 + 0.7 lysoPC a C16:0 - 0.451 age \| \| --- \| \| The optimal cut-off point for the above equation is 0.58. \| | | | | | |
